# Real-time evaluation of optic nerve sheath diameter (ONSD) in awake neurosurgical patients

**DOI:** 10.1186/2197-425X-3-S1-A608

**Published:** 2015-10-01

**Authors:** N Weidner, H Bomberg, S Antes, A Meiser, T Volk, HV Groesdonk

**Affiliations:** Department of Anesthesiology, Critical Care and Pain Medicine, University Hospital Homburg/Saar, Homburg, Germany; Department of Neurosurgery, University Hospital Homburg/Saar, Homburg, Germany

## Introduction

Ultrasound measurement of the optic nerve sheath diameter (ONSD) has been utilized as an indirect assessment of intracranial pressure (ICP). Currently, the normal ONSD is believed to be < 5 mm, whereas diameters above 6 mm are considered to reflect a clinically significant increase in ICP. Despite these standard values, the ONSD has never been compared with simultaneously measured ICP values in awake, spontaneously breathing patients.

## Objectives

In this prospective study, we investigated the correlation between ONSD and the simultaneously measured ICP by means of an intraparenchymatic P-Tel probe in awake, spontaneously breathing patients.

## Methods

ICP was measured continuously in 15 patients by means of an intraparenchymatic P-Tel probe. Additionally, ONSD was measured in all patients by the readily available LOGIQ e Ultrasound machine from General Electric (GE Healthcare, Little Chalfont, UK) with a high-frequency 7.5-10-MHz or higher linear array ultrasound transducer. The ONSD was measured 3 mm behind the optic disc in both transverse and sagittal planes. The average of the three measurements was recorded and compared with the ICP. Additionally, ONSD of patients with an ICP of 1-10 mmHg (n=6) were compared with ONSD of patients suffering from an ICP of 11-20 mmHg (n=9).

## Results

In all patients and for both eyes, the ONSD correlated well with the simultaneously measured ICP (Pearson R = 0.77 - R = 0.88). Comparison of patients with normal and increased ICP revealed that the ONSD measurement accurately predicts an elevated ICP with an optimal cut-off value of 5.05 mm (AUC of 0.93, sensitivity 92%, and specificity 89%, p < 0.001).

## Conclusions

This study underlines the usefulness of ONSD measurement to accurately predict increased ICP, especially in awake, spontaneously breathing patients.

